# Musculoskeletal Model Personalization Affects Metabolic Cost Estimates for Walking

**DOI:** 10.3389/fbioe.2020.588925

**Published:** 2020-11-26

**Authors:** Marleny M. Arones, Mohammad S. Shourijeh, Carolynn Patten, Benjamin J. Fregly

**Affiliations:** ^1^Department of Mechanical Engineering, Rice University, Houston, TX, United States; ^2^Department of Physical Medicine and Rehabilitation, University of California, Davis, Davis, CA, United States

**Keywords:** metabolic cost, musculoskeletal model, EMG-driven model, model personalization, stroke, gait

## Abstract

Assessment of metabolic cost as a metric for human performance has expanded across various fields within the scientific, clinical, and engineering communities. As an alternative to measuring metabolic cost experimentally, musculoskeletal models incorporating metabolic cost models have been developed. However, to utilize these models for practical applications, the accuracy of their metabolic cost predictions requires improvement. Previous studies have reported the benefits of using personalized musculoskeletal models for various applications, yet no study has evaluated how model personalization affects metabolic cost estimation. This study investigated the effect of musculoskeletal model personalization on estimates of metabolic cost of transport (CoT) during post-stroke walking using three commonly used metabolic cost models. We analyzed walking data previously collected from two male stroke survivors with right-sided hemiparesis. The three metabolic cost models were implemented within three musculoskeletal modeling approaches involving different levels of personalization. The first approach used a scaled generic OpenSim model and found muscle activations via static optimization (SOGen). The second approach used a personalized electromyographic (EMG)-driven musculoskeletal model with personalized functional axes but found muscle activations via static optimization (SOCal). The third approach used the same personalized EMG-driven model but calculated muscle activations directly from EMG data (EMGCal). For each approach, the muscle activation estimates were used to calculate each subject’s CoT at different gait speeds using three metabolic cost models (Umberger et al., 2003; Bhargava et al., 2004; Umberger, 2010). The calculated CoT values were compared with published CoT data as a function of walking speed, step length asymmetry, stance time asymmetry, double support time asymmetry, and severity of motor impairment (i.e., Fugl-Meyer score). Overall, only SOCal and EMGCal with the Bhargava metabolic cost model were able to reproduce accurately published experimental trends between CoT and various clinical measures of walking asymmetry post-stroke. Tuning of the parameters in the different metabolic cost models could potentially resolve the observed CoT magnitude differences between model predictions and experimental measurements. Realistic CoT predictions may allow researchers to predict human performance, surgical outcomes, and rehabilitation outcomes reliably using computational simulations.

## Introduction

Metabolic cost has been used to evaluate human performance during daily activities such as walking (Waters and Mulroy, 1999; Donelan et al., 2002, 2008; Mian et al., 2006; Long and Srinivasan, 2013) and athletic activities such as running (Roberts et al., 1998; Chang and Kram, 1999; Teunissen et al., 2007; Franz et al., 2012; Long and Srinivasan, 2013) and cycling (Davies, 1980; Gnehm et al., 1997; Neptune and Van Den Bogert, 1997; McDaniel et al., 2002; van der Woude et al., 2008). Metabolic cost is defined as the energy consumed by the body during a given activity, a quantity that has also been adopted as a metric to evaluate the design or operational settings of assistive devices (Malcolm et al., 2013; Collins et al., 2015; Galle et al., 2017). Additional applications for which knowledge of metabolic cost is useful include: prescription of training intensities (American College of Sports Medicine, 2000), advancement of geriatric medicine (Mian et al., 2006; Canavan et al., 2009; Corbett et al., 2017), treatment of clinical gait disorders (Waters and Mulroy, 1999), and monitoring of energy intake and expenditure in obese patients (Brychta et al., 2010). Various methods exist to measure metabolic cost, with the two most popular being indirect and direct calorimetry. Direct calorimetry measures metabolic cost using a calorimeter and is the most accurate method. However, its usage is limited due to the cost of dedicated equipment and the need for specific expertise to acquire and interpret the data. In contrast, indirect calorimetry estimates metabolic cost by measuring respiratory gases influenced by the body’s metabolism (Lam and Ravussin, 2016). Although indirect calorimetry is more affordable than direct calorimetry, the trade-off is reduced accuracy. Regardless of the method, the various applications of metabolic cost measurement often require subjects to walk repeatedly for long periods of time, limiting the participation of subjects with severe impairments or who quickly fatigue (Markovitz et al., 2004; Awad et al., 2017).

With advances in computational biomechanics, musculoskeletal models incorporating metabolic cost models have emerged as tools to estimate metabolic cost. These tools have been used to predict human movement and response to mechanical interventions (Uchida et al., 2016; Falisse et al., 2019). Although the creation and calibration of musculoskeletal models involves high computational cost, once a subject-specific model is created, it is easier, faster, and cheaper to evaluate various treatment options *in silico*. Specifically, within the field of exoskeleton design, musculoskeletal models can eliminate the time and expense of iteratively designing and building physical prototypes. Uchida et al. (2016) used the OpenSim simulation framework (Seth et al., 2018) to optimize the design of an assistive device intended to reduce the metabolic cost of running. Dembia et al. (2017) simulated an ideal assistive device to minimize the metabolic cost of several individuals walking with heavy loads. Fey et al. (2012) developed an optimization to identify an optimal prosthetic foot stiffness to minimize metabolic cost for amputee walking. Miller et al. (2013) used direct collocation optimal control to predict a gait pattern that reduced metabolic cost while minimizing peak axial knee contact force. By predicting the factors, designs, or movements that minimize metabolic cost, these studies highlight the potential benefits of combining musculoskeletal and metabolic cost models. However, for these predictions to be used for practical applications, they need to be validated against experimental measurements. Studies that have performed a direct comparison between measured and predicted metabolic cost have reported noticeable differences depending on the metabolic cost model chosen (Miller, 2014; Koelewijn et al., 2019), emphasizing the need to improve either the accuracy of existing metabolic cost models, the fidelity of the associated musculoskeletal models, or both.

Previous studies that estimated metabolic cost during walking have focused on using scaled generic musculoskeletal models. However, several studies have reported that personalization of anatomical and physiological characteristics of a musculoskeletal model can influence prediction of muscle forces, joint moments, and novel movements, factors that also play a role in metabolic cost calculations. Reinbolt et al. (2007) found that personalization of the joint functional axes is important for obtaining reliable inverse dynamic joint moments. Several studies (e.g., Lloyd and Besier, 2003; Buchanan et al., 2005; Shao et al., 2009; Sartori et al., 2012; Meyer et al., 2017) reported large improvements in joint moment matching when muscle force-generating properties were personalized using an electromyographic (EMG)-driven model. Gerus et al. (2013) demonstrated that personalizing knee geometry and corresponding muscle-tendon model parameter values resulted in improved predictions of knee contact force compared to predictions generated by a generic model. Since muscle force estimates depend on joint moments, and the production of muscle forces and activations depends on muscle-tendon model parameters, the confounding effect of model personalization may also affect metabolic cost estimates. However, to the best of the authors’ knowledge, no study to date has explored this possibility.

This study evaluated the influence of musculoskeletal model personalization on metabolic cost estimates of walking post-stroke. To evaluate the physical realism of different metabolic cost modeling methods, we compared metabolic cost estimates to trends reported in the literature for individuals post-stroke. Finley and Bastian (2017) reported a decrease in metabolic cost as walking speed increased and severity of motor impairment decreased. For the same subject population, Finley et al. (2015) and Finley and Bastian (2017) also reported that metabolic cost increased as asymmetries in step length, stance time, and double support time increased. To compare with these experimental trends, the present study calculated metabolic cost estimates using extensive gait data sets collected previously from two individuals post-stroke for whom metabolic cost measurements were not available. Our goal was to determine whether any combination of musculoskeletal modeling method and metabolic cost modeling method could reproduce all five physically realistic metabolic cost trends observed in the literature. Since metabolic cost has been adopted as a tool in various scientific, clinical, and engineering fields to evaluate human performance, surgical outcomes, and rehabilitation outcomes, the results of this study may be useful for identifying which modeling methods are most likely to predict physically realistic metabolic cost estimates.

## Materials and Methods

### Experimental Data and Data Processing

Experimental walking data collected from two male stroke survivors–one high functioning and one low functioning–were used as inputs to the metabolic cost analyses (see Table 1 for subject characteristics). Data collected from both subjects included marker-based motion, ground reaction, and surface and fine-wire EMG data (Table 2, 16 channels per leg). The data were collected at different walking speeds using a split-belt instrumented treadmill with belts tied. Walking speeds ranged from slower than self-selected to a maximum comfortable speed. For the high functioning subject, the speed range was from 0.4 to 0.8 m/s in increments of 0.1 m/s, while for the low functioning subject, the speed range was from 0.35 to 0.65 m/s also in increments of 0.1 m/s. Additional experimental data were also collected for static and isolated joint motion trials. A static standing trial was collected for model scaling purposes. Isolated joint motion trials were collected for each hip, knee, and ankle for purposes of personalizing the model’s lower body joint centers and functional axes. All functional axes for the joint of interest were exercised during each isolated joint motion trial (Reinbolt et al., 2005, 2008).

**TABLE 1:** Clinical characteristics of study participants.

Gender	Age	Paretic Limb	Lower Limb Fugl-Meyer	Self-Selected Speed (m/s)
M	79	R	32	0.5
M	62	R	25	0.35
				

**TABLE 2:** Muscle groups from which a surface or fine-wire (*) EMG signal was recorded.

High Functioning Subject	Low Functioning Subject
*Adductor Longus	*Adductor Longus
Gluteus Maximus	Gluteus Maximus
Gluteus Medius	Gluteus Medius
*Iliopsoas	*Iliopsoas
Semimembranosus	Semimembranosus
Biceps Femoris Long Head	Biceps Femoris Long Head
Rectus Femoris	Tensor Fasciae Latae
Vastus Lateralis	Rectus Femoris
Vastus Medialis	Vastus Lateralis
Gastrocnemius Medialis	Vastus Medialis
*Tibialis Anterior	Gastrocnemius Lateralis
Tibialis Posterior	Gastrocnemius Medialis
Peroneus Longus	Tibialis Anterior
Soleus	*Tibialis Posterior
*Extensor Digitorum Longus	Peroneus Longus
*Flexor Digitorum Longus	Soleus
	

The experimental data were processed using standard methods. The ground reaction and marker motion data were low-pass filtered using a fourth-order zero-phase lag Butterworth filter with a cut-off frequency of 7/*t*_*f*_ Hz (Hug, 2011), where *t*_*f*_ is the period of the gait cycle being processed (Meyer et al., 2017). On average, this variable cut-off frequency caused data collected at a normal walking speed to be filtered at approximately 6 Hz. The EMG data were high-pass filtered (40 Hz), demeaned, rectified, and low-pass filtered (3.5/*t*_*f*_ Hz) using a fourth-order zero-phase lag Butterworth filter. EMG amplitudes for each muscle were normalized to the maximum value over all trials and resampled to 101 time points per gait cycle, as described in Meyer et al. (2017).

### Musculoskeletal Model

A generic full-body OpenSim musculoskeletal model (Rajagopal et al., 2016; Seth et al., 2018) served as the starting point for all three metabolic cost analyses. The generic model used for both subjects started with 40 Hill-type muscle-tendon actuators per leg and 37 degrees of freedom (DOFs), including: 3 DOF hip joints, 1 DOF knee joints, 2 DOF ankle joints. For the high functioning subject, six muscles without related EMG data were eliminated (extensor hallucis longus, flexor hallucis longus, gracilis, piriformis, sartorius, tensor fascia latae). For the low functioning subject, seven muscles without related EMG data were eliminated (extensor digitorum longus, flexor digitorum longus, extensor hallucis longus, flexor hallucis longus, gracilis, piriformis, sartorius). The remaining muscles actuated hip flexion-extension, hip adduction-abduction, hip internal-external rotation, knee flexion-extension, ankle flexion-extension, and ankle inversion–eversion on each leg.

### Joint Model Personalization

Personalization of the joint functional axes for the hip, knee, and ankle of each leg was performed by following a two-step process. First, the geometry of the generic OpenSim model was scaled to match the dimensions of each subject using the OpenSim Scale Model tool and the static standing trial data. Second, marker positions and functional axes of the model’s lower body joints were personalized as described in Reinbolt et al. (2005, 2008), Meyer et al. (2016), and Sauder et al. (2019). The personalization process involved using non-linear optimization to adjust the positions and orientations of the model’s lower body joints and marker triads placed on the body segments. The cost function minimized the sum of squares of errors between the experimental and model marker positions from all isolated joint motion trials and one walking trial analyzed together. The optimization process was performed using Matlab’s *lsqnonlin* algorithm, which iteratively ran OpenSim Inverse Kinematics analyses to calculate marker location errors.

### Muscle-Tendon Model and Geometry Personalization

Experimental data from ten gait trials collected at each available walking speed were used to calibrate an EMG-driven model of both legs for each subject. Before performing EMG-driven model calibration, we analyzed marker data from each gait trial using the OpenSim Inverse Kinematics tool to generate joint angle trajectories. The OpenSim Inverse Dynamics tool was then used to calculate the joint moments produced by muscle forces. Next, a surrogate model of each subject’s musculoskeletal geometry was generated to allow the EMG-driven model to modify musculoskeletal geometry (Meyer et al., 2016, 2017). The surrogate model was developed by sampling a wide range of joint angle combinations for the lower limbs using a Latin hypercube design. Muscle-tendon lengths and moment arms for each muscle were then calculated using the OpenSim Muscle Analysis tool. Linear regression using least squares was used to fit muscle-tendon lengths and moment arms simultaneously as related polynomial functions of the corresponding joint angles actuated by each muscle. Each muscle-tendon moment arm polynomial was defined to be the first derivative of the related muscle-tendon length polynomial with respect to the associated joint angle (An et al., 1984). Muscle-tendon velocity was defined as the first derivative of the resulting muscle-tendon length polynomial with respect to time.

To personalize the model’s muscle-tendon force-generating properties, we allowed our EMG-driven model calibration process to modify three types of parameters: EMG-to-activation parameters (electromechanical delays, activation dynamics time constants, activation non-linearization shape factors, and EMG scale factors), Hill-type muscle-tendon model parameters (optimal muscle fiber lengths and tendon slack lengths), and surrogate musculoskeletal geometry parameters. Using Matlab’s *fmincon* algorithm with sequential quadratic programming, we adjusted the model parameter values to best match calculated experimental inverse dynamic joint moments and published passive joint moments (Silder et al., 2007). In addition, penalty terms were added to the cost function to discourage substantial divergence of model parameter values from their original values (Supplementary Tables S1, S2). A detailed description of our EMG-driven modeling approach can be found in Meyer et al. (2017) as well as in the Supplementary Material.

### Metabolic Cost Analysis

**FIGURE 1:**
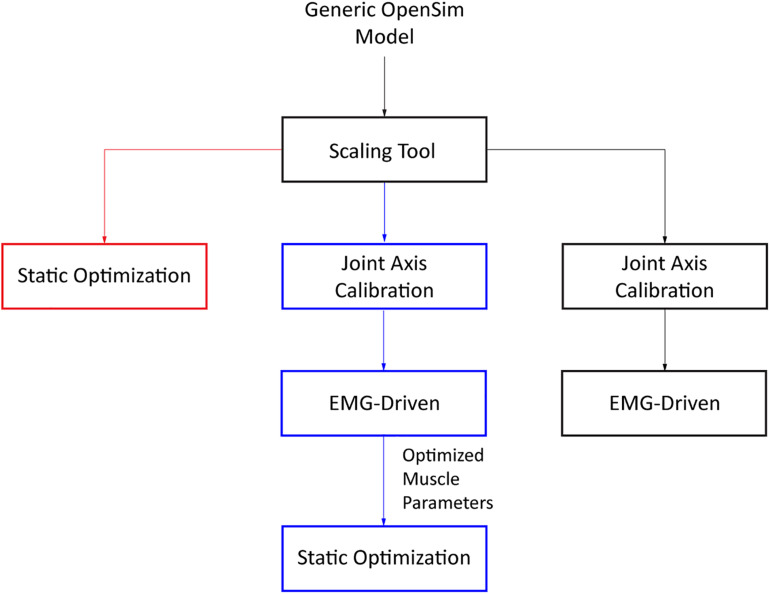
Flowchart of the three different approaches used to obtain estimates of muscle activations (SOGen: red, SOCal: blue, EMGCal: black).

To evaluate the extent to which model personalization affects estimated metabolic cost, we developed three musculoskeletal models for each subject with varying levels of personalization. The least personalized model was a scaled generic OpenSim model where muscle activations were calculated via static optimization using quadratic programming (SOGen) (Supplementary Figure S1; Shourijeh and Fregly, 2020). The intermediate personalized model used the subject’s calibrated EMG-driven model but with muscle activations found via static optimization (SOCal) (Supplementary Figure S2). The most personalized model again used the subject’s calibrated EMG-driven model but with muscle activations calculated from the subject’s experimental EMG data (EMGCal) (Figure 1 and Supplementary Figure S3). The muscle activations found by each approach were used to calculate each subject’s cost of transport (CoT in J/m/kg) for different gait speeds using metabolic cost models published by Umberger et al. (2003) (U03), Umberger (2010) (U10), and Bhargava et al. (2004) (B04). CoT is defined as the metabolic cost expended to move a unit of body mass a unit of distance. These three metabolic cost models were chosen due to their popularity, where all three models are a function of work and heat rate due to muscle activation, muscle shortening and lengthening, and maintenance of muscle contraction. The key difference between these models is the assignment of negative (B04) or positive (U10 and U03) heat rate during muscle lengthening, along with the inclusion (U03 and B04) or exclusion (U10) of eccentric contraction work rate in the metabolic cost calculations.

To evaluate the physical realism of each musculoskeletal model/metabolic cost model combination, we identified five experimental trends in the literature for how CoT varies as a function of other clinically relevant quantities for individuals post-stroke. The first three quantities were step length asymmetry, stance time asymmetry, and double-support time asymmetry, all of which have been reported to increase as the CoT increases (Finley and Bastian, 2017). The two other quantities were walking speed and Fugl-Meyer score, which have been observed to decrease as the CoT increases. Asymmetries in step length, stance time, and double-support time between the paretic and non-paretic legs were calculated as specified in Finley et al. (2015) and Finley and Bastian (2017), where paretic leg values were subtracted from the non-paretic leg values and then absolute values taken. Step length asymmetry was computed as the difference between distance between the non-paretic foot and the pelvis during heel strike of the non-paretic leg and distance between the paretic foot and the pelvis at heel strike of the paretitc leg (defined as the difference in step position in Finley et al., 2015 and Finley and Bastian, 2017). Stance time asymmetry was computed as the difference between duration from heel strike to toe-off of the non-paretic leg and duration from heel strike to toe-off of the paretic leg. Double-support time asymmetry was computed as the difference between duration from heel strike of the paretic leg to toe-off of the non-paretic leg and duration from heel strike of the non-paretic leg to toe-off of the paretic leg.

We performed statistical analyses to evaluate whether trends in CoT as a function of the five quantities described above were different between each musculoskeletal model/metabolic cost model combination and the experimental data published in Finley and Bastian (2017). The selected statistical analysis was analysis of covariance, which compared the slopes and y-intercepts of regression models that fitted model predictions and experimental measurements. To provide a fair comparison with the experimental data published in Finley and Bastian (2017), we cropped the experimental data points to within ±0.05 m/s of the maximum and minimum speeds used in our study. This criterion was chosen to maximize the number of experimental data points retained from Finley and Bastian (2017) study while being consistent with the 0.05 m/s speed interval present in our study. To examine the relationship between Fugl-Meyer score and CoT, we first calculated the average CoT value across all speeds and trials per subject. These values were then compared to the average CoT values published in Finley and Bastian (2017) for a subset of subjects whose Fugl-Meyer scores were within ±3 of our high and low functioning subjects.

## Results

**FIGURE 2:**
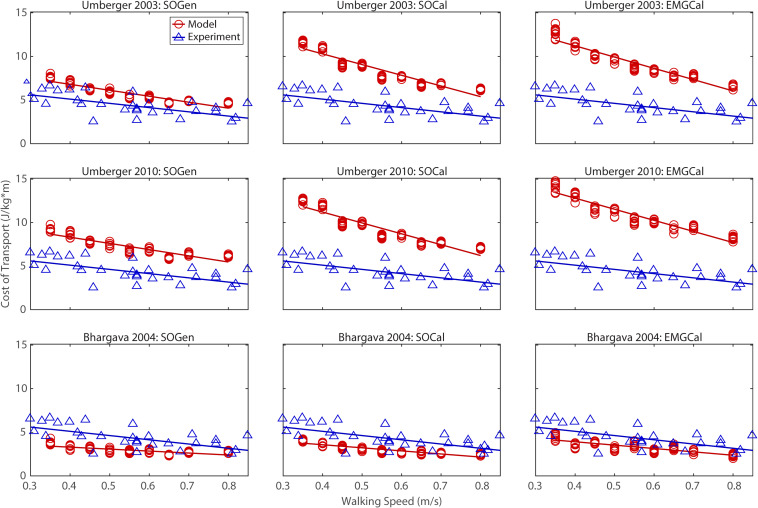
Comparison of model predicted (red circles) and experimentally measured (blue triangles) cost of transport as a function of walking speed. Model predictions include all possible combinations of three musculoskeletal models possessing different levels of personalization (columns) and three metabolic cost models (rows). Experimental measurements were obtained from Finley and Bastian (2017).

**FIGURE 3:**
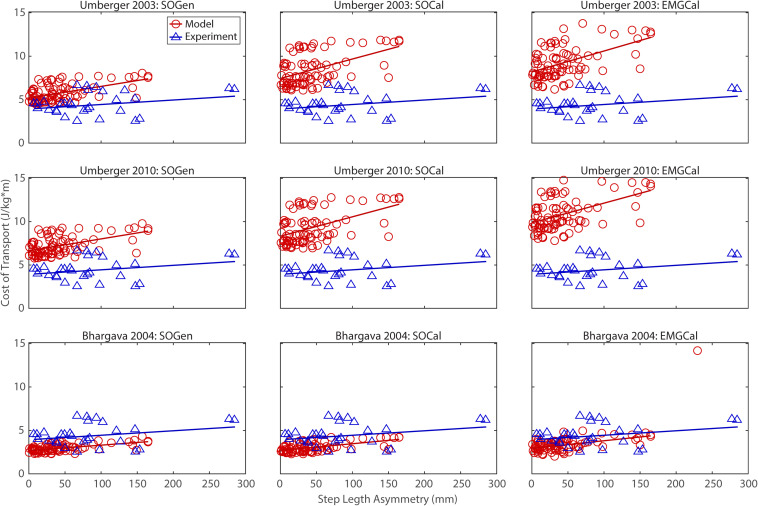
Comparison of model predicted (red circles) and experimentally measured (blue triangles) cost of transport as a function of step length asymmetry between paretic and non-paretic legs. See Figure 2 caption for additional details.

**FIGURE 4:**
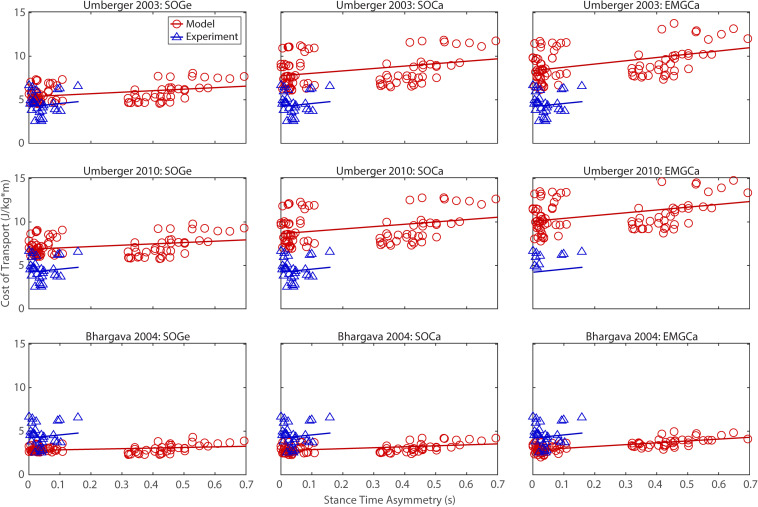
Comparison of model predicted (red circles) and experimentally measured (blue triangles) cost of transport as a function of stance time asymmetry between paretic and non-paretic legs. See Figure 2 caption for additional details.

**FIGURE 5:**
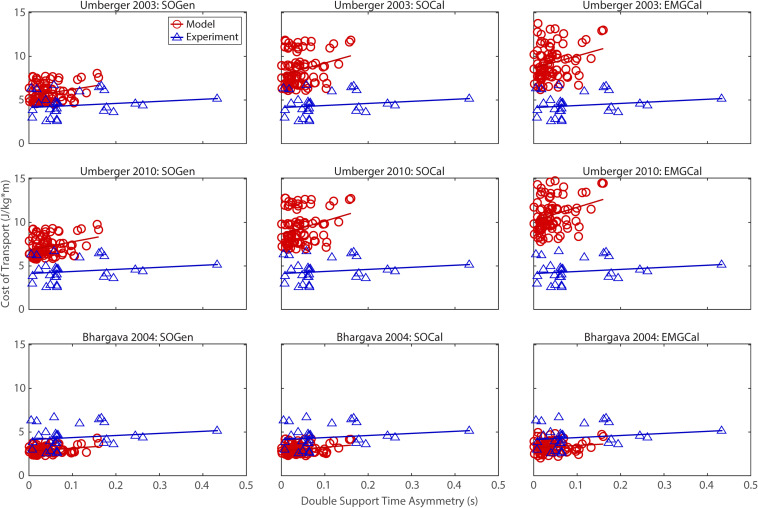
Comparison of model predicted (red circles) and experimentally measured (blue triangles) cost of transport as a function of double support time asymmetry between paretic and non-paretic legs. See Figure 2 caption for additional details.

The ability to predict CoT trends consistent with experimental measurements varied across the nine modeling combinations (Table 3 and Figures 2–5). Overall, only the Bhargava et al. (2004) metabolic cost model predicted slopes of CoT versus walking speed, step length asymmetry, stance time asymmetry, and double support time asymmetry regression lines that were statistically similar to those measured experimentally in Finley and Bastian (2017). Within the Bhargava metabolic cost model, *p*-values tended to increase as the level of musculoskeletal model personalization increased, with the EMGCal musculoskeletal model generally exhibiting the largest *p*-values and thus the greatest statistical similarity to experimental measurements. In contrast, the Umberger et al. (2003) and Umberger (2010) metabolic cost models predicted slopes that were statistically similar to those measured experimentally for only stance time asymmetry and double support time asymmetry. Within the two Umberger metabolic cost models, *p*-values tended to be comparable across the three levels of musculoskeletal model personalization.

**TABLE 3:** Analysis of covariance *p*-values comparing slopes of CoT versus walking speed, step length asymmetry, stance time asymmetry, and double support time asymmetry regression lines between the nine model predictions and experimental measurements from Finley and Bastian (2017).

	Umberger et al., 2003			Umberger, 2010			Bhargava et al., 2004		
	SOGen	SOCal	EMGCal	SOGen	SOCal	EMGCal	SOGen	SOCal	EMGCal
Walking Speed	0.018	0.000	0.000	0.013	0.000	0.000	0.001	**0.073**	**0.213**
Step Length Asymmetry	0.030	0.004	0.002	0.035	0.005	0.005	**0.701**	**0.367**	**0.245**
Stance Time Asymmetry	**0.722**	**0.920**	**0.983**	**0.713**	**0.915**	**0.966**	**0.420**	**0.514**	**0.686**
Double Support Time Asymmetry	**0.171**	**0.061**	**0.071**	**0.094**	**0.050**	0.045	**0.701**	**0.601**	**0.975**

*Statistically similar slopes (*p* > 0.05) are shown in bold.*

In contrast, the ability to predict CoT magnitudes consistent with experimental measurements was weak for all nine modeling combinations. Specifically, none of the nine modeling combinations predicted y-intercepts of CoT versus speed, step position asymmetry, stance time asymmetry, or double support time asymmetry regression lines that were statistically similar to those measured experimentally in Finley and Bastian (2017) (all *p*-values < 0.05). However, when absolute values of y-intercept differences were calculated (Table 4), the Bhargava et al. (2004) metabolic cost model tended to have the smallest differences, consistent with possessing the smallest regression line offsets relative to the experimental regression lines (Figures 2–5). Furthermore, within the Bhargava metabolic cost model, y-intercept differences generally decreased as the level of musculoskeletal model personalization increased, with the EMGCal musculoskeletal model again exhibiting the greatest similarity to experimental measurements.

**TABLE 4:** Absolute differences in regression line y-intercepts of CoT versus walking speed, step length asymmetry, stance time asymmetry, and double support time asymmetry between the nine model predictions and experimental measurements from Finley and Bastian (2017).

	Umberger et al., 2003			Umberger, 2010			Bhargava et al., 2004		
	SOGen	SOCal	EMGCal	SOGen	SOCal	EMGCal	SOGen	SOCal	EMGCal
Walking Speed	2.48	8.14	9.34	4.12	9.15	10.86	2.86	2.07	1.59
Step Length Asymmetry	1.25	3.52	4.27	2.72	4.40	5.94	1.24	1.23	0.98
Stance Time Asymmetry	1.14	3.55	4.14	2.65	4.45	5.88	1.42	1.44	1.39
Double Support Time Asymmetry	1.30	3.70	4.53	2.71	4.55	6.09	1.32	1.26	0.91
									

When mean CoT across speeds was compared between each subject used in our study and subjects with comparable Fugl-Meyer scores in Finley and Bastian (2017), the U03-SOGen, B04-SOCal, and B04-EMGCal model combinations exhibited the best ability to predict CoT as a function of Fugl-Meyer score (Table 5). Specifically, for both subjects, U03-SOGen, B04-SOCal, and B04-EMGCal predicted mean CoT values within two standard deviations of the mean CoT value calculated for corresponding subjects in Finley and Bastian (2017). The B04-SOGen mean CoT value for the high functioning subject was also within two standard deviations of Finley and Bastian’s subjects. In addition, all nine modeling combinations predicted a higher mean CoT value for the low functioning subject than for the high functioning subject, in agreement with (Finley and Bastian, 2017).

**TABLE 5:** Comparison of cost of transport between the nine modeling combinations and the experimental measurements reported in Finley and Bastian (2017) as a function of Fugl-Meyer score.

High-Functioning Subject										
		Umberger et al., 2003			Umberger, 2010			Bhargava et al., 2004		
CoT	Finley	SOGen	SOCal	EMGCal	SOGen	SOCal	EMGCal	SOGen	SOCal	EMGCal
Mean	3.61	5.56	8.13	8.73	7.10	9.04	10.46	2.89	2.89	2.92
Std Dev	1.45	0.88	1.71	1.67	0.93	1.76	1.71	0.27	0.46	0.43

**Low-Functioning Subject**										

Mean	5.1	5.98	8.74	9.82	7.38	9.60	11.30	3.03	3.19	3.77
Std Dev	1.02	1.13	1.89	1.95	1.26	1.94	1.99	0.54	0.60	0.53

*The high functioning subject (top) possessed a Fugl-Meyer score of 32, while low functioning subject (bottom) had a Fugl-Meyer score of 25. Experimental measurements used for comparison were taken from Finley subjects with similar Fugl-Meyer scores.*

## Discussion

This study evaluated the effect of musculoskeletal model personalization on metabolic cost estimates for walking post-stroke obtained using three published metabolic cost models: Umberger et al. (2003) (U03), Umberger (2010) (U10), and Bhargava et al. (2004) (B04). These three metabolic cost models were implemented within three musculoskeletal models incorporating varying levels of personalization: scaled generic musculoskeletal models with muscle activations found by static optimization (SOGen), calibrated EMG-driven musculoskeletal models with muscle activations found by static optimization (SOCal), and calibrated EMG-driven musculoskeletal models with muscle activations found from experimental EMG data (EMGCal). These nine modeling combinations were investigated using published walking data collected from two individuals post-stroke, and trends in estimated CoT as a function of walking speed, step length asymmetry, stance time asymmetry, and double support time asymmetry were compared with published experimental data collected from individuals post-stroke (Finley and Bastian, 2017). All nine modeling combinations predicted the correct positive and negative correlations between CoT and the four selected quantities (excluding Fugl-Meyer score) as observed in Finley and Bastian’s post-stroke population. However, for the four other quantities, only the personalized musculoskeletal models (SOCal and EMGCal) paired with the Bhargava et al. (2004) metabolic cost model produced slopes that were statistically similar to those calculated from Finley and Bastian’s experimental data. Specifically, B04-EMGCal exhibited the strongest similarities to Finley and Bastian (2017) as noted by *p*-values that were all greater than 0.2 (Table 3). Although B04-SOCal and B04-EMGCal were the only modeling combinations that produced slopes for CoT versus speed regression lines that were statistically similar to Finley and Bastian (2017), B04-SOCal’s *p*-value of 0.073 approached a statistically significant difference. In addition, between these two modeling combinations, B04-EMGCal produced y-intercepts for CoT versus speed regression lines that were closest to Finley and Bastian (2017). While we could not investigate thoroughly the relationship between CoT and Fugl-Meyer score due to having only two subjects in our study, B04-EMGCal was still one of the best model combinations for consistency with (Finley and Bastian, 2017). These findings suggest that a calibrated EMG-driven musculoskeletal model combined with the Bhargava et al. (2004) metabolic cost model may provide the best CoT estimates during walking for individuals post-stroke.

In general, *p*-values assessing statistical differences between predicted and measured (Finley and Bastian, 2017) regression slopes of CoT versus walking speed, step length asymmetry, stance time asymmetry, and double support time asymmetry increased as the level of musculoskeletal model personalization increased from B04-SOGen to B04-SOCal and B04-EMGCal. Similarly, absolute differences between predicted and measured regression y-intercepts decreased as the level of musculoskeletal model personalization increased from B04-SOGen to B04-SOCal and B04-EMGCal. This pattern was not observed across musculoskeletal model personalization levels for the Umberger et al. (2003) and Umberger (2010) metabolic cost models. For these models, the magnitudes of predicted CoT values were almost twice as large as the published CoT averages. These observations can be explained by looking at the RMSE errors between muscle activations produced by static optimization to those obtained from the EMG-Driven model (Supplementary Table S3). On average, SOGen produced RMSE errors of 0.13 (±0.10) for the high functioning patient and 0.14 (±0.12) for the low functioning patient. SOCal produced RMSE errors of 0.10 (±0.09) for the high functioning subject and 0.11 (±0.10) for the low functioning subject. Thus, SOGen produced slightly larger RMSE errors than did SOCal. However, due to minimization of muscle activation inherent to static optimization, SOGen and SOCal both struggled to match the larger muscle activations produced in the EMG-driven model. This difference in magnitude is amplified in both of Umberger’s models due to the activation dependent scaling factors associated to the calculation of the maintenance heat rate and the shortening/lengthening heat rate.

Apart from differences in muscle activations, differences in joint moment matching also existed between static optimization and EMG-driven modeling (Supplementary Table S4). On average, due to the omission of calibrated joint functional axes, SOGen produced RMSE errors of 1.52 ± 1.08 Nm for the high functioning subject and 1.89 ± 2.48 Nm for the low functioning subject. In contrast, EMGCal produced RMSE errors of 4.78 ± 1.91 Nm for the high functioning subject and 4.55 ± 2.09 Nm for the low functioning subject. Although the RMSE errors were lower for SOGen than for EMGCal, the average errors in joint moment matching for EMGCal were within the ranges reported by Meyer et al. (2017), even though the present study matched an additional moment at the hip (i.e., internal-external rotation).

For all three levels of musculoskeletal model personalization, the Bhargava et al. (2004) metabolic cost model tended to slightly underestimate the experimental CoT values reported in Finley and Bastian (2017), while the Umberger et al. (2003) and Umberger (2010) metabolic cost models tended to overestimate the experimental values. The same trends were observed in Miller (2014), which compared the performance of B04 and U10, among other models. In contrast to studies like Miller (2014) and Ong et al. (2019), which used a musculoskeletal model combined with the U03 metabolic cost model and predicted CoT values within the range of 2 to 8 J/Kg.m, the U03-SOCal, U03-EMGCal, U10-SOCal, and U10-EMGCal model combinations in our study produced CoT values ranging from 7 to 15 J/Kg.m. This magnitude difference was unexpected but may be due to the minimization of a metabolic cost term within the optimization cost function used for estimating muscle activations in these two previous studies. Furthermore, in contrast to the two studies mentioned above, our study used calibrated EMG-driven models to calculate CoT. The absence of muscle activation minimization may help explain why our EMGCal musculoskeletal model paired with any metabolic cost model produced CoT estimates that were larger than those produced by SOGen and SOCal, as static optimization is known to produce the lowest possible level of muscle co-contraction while still matching the joint moment constraints (Heintz and Gutierrez-Farewik, 2007; Shourijeh and Fregly, 2020).

Although all modeling combinations correctly predicted the positive or negative trends in published experimental CoT data, not all combinations resulted in regression slopes that were statistically similar to those found in Finley and Bastian’s (2017) experimental data. This finding is similar to a recent study by Koelewijn et al. (2019), where several metabolic cost models (including U03 and B04) were shown to predict the correct trends for CoT as a function of walking slope or gait speed in accordance with trends found in the literature (Margaria, 1968). In addition, Koelewijn et al. (2019) found that B04 tended to underestimate CoT values in comparison to experimental values, consistent with our findings.

To improve CoT predictions further, researchers should consider optimizing the various parameters within metabolic cost models, such as maximum muscle stress. As reported in Russell Esposito and Miller (2018), decreasing the maximum muscle stress value increases metabolic cost and vice versa, which could help reduce magnitude discrepancies in CoT predictions. Interestingly, when (Falisse et al., 2019) used the Bhargava et al. (2004) metabolic cost model with different values of maximum muscle stress for different muscles, they obtained reasonable agreement between model predicted and experimentally measured CoT values. Alternatively, one could use a single scale factor to calibrate metabolic cost models as reported in Michaud et al. (2019), who used both B04 and U03 to find good agreement between model predicted and published experimentally measured CoT values.

The most significant limitation of this study was the absence of experimental metabolic cost data for evaluating our model-based estimates. This limitation makes it difficult to determine the accuracy of our modeling methods. Furthermore, none of the musculoskeletal models used in our analyses included muscles to actuate the upper body. Consequently, the calculated CoT for all nine model combinations may be an underestimate. However, since Finley and Bastian’s data set was collected from subjects who were allowed to use handrails, their measurements may also be underestimates. In addition, we were limited to studying only two subjects, since extensive EMG data sets were required to perform the study. In contrast, the data reported in Finley and Bastian (2017) were based on 15 individuals who were more than 6 months post-stroke (58 ± 14 years old) with an average Fugl-Meyer score of 23 ± 6 and an average self-selected speed of 0.43 m/s ± 0.3. Both of our subjects were more than 6 months post-stroke, and the age and Fugl-Meyer score of the high functioning subject exceeded one standard deviation above the average population metrics found in Finley and Bastian (2017) (Table 1). Therefore, our high functioning subject may not have been as well represented in the published data from Finley and Bastian.

## Conclusion

In conclusion, this study investigated the effect of musculoskeletal model personalization on estimated metabolic cost for walking post-stroke as calculated using three published metabolic cost models. Previously collected walking data from two stroke survivors were used to analyze correlations between estimated CoT and various variables commonly associated with gait asymmetry. Although all modeling combinations exhibited the correct positive and negative correlations observed in Finley and Bastian (2017), only B04-SOCal and B04-EMGCal produced statistically similar regression slopes to those found in Finley and Bastian for walking speed and all gait asymmetry variables (excluding Fugl-Meyer score). While the regression y-intercepts were not statistically similar to those found in Finley and Bastian (2017), B04-EMGCal produced CoT estimates that were closest in magnitude to those calculated from Finlay and Bastian’s data. Our results suggest that use of a personalized EMG-driven model paired with Bhargava’s metabolic cost model may improve prediction of CoT during walking for individuals post-stroke. Since metabolic cost can be used as a tool to gauge physical exertion, the results of this study may impact scientific, clinical, and engineering fields that target minimization of metabolic cost through surgical or rehabilitation methods.

## Data Availability Statement

The experimental data used to perform this study are available at https://simtk.org/projects/cot-personalize.

## Ethics Statement

All experimental procedures were approved by the University of Florida Health Science Center Institutional Review Board (IRB-01), and the subject provided written informed consent prior to participation.

## Author Contributions

CP and BF performed the experiments. MA performed all model personalization tasks, prepared the figures, and drafted the manuscript. MA and MS analyzed the data. MA, MS, and BF interpreted the results of analyses. All authors revised the manuscript.

## Conflict of Interest

The authors declare that the research was conducted in the absence of any commercial or financial relationships that could be construed as a potential conflict of interest.
