# Supplementary material for: Musculoskeletal Model Personalization Affects Metabolic Cost Estimates for Walking
Source: Front Bioeng Biotechnol. 2020 Nov 26;8:588925. doi: 10.3389/fbioe.2020.588925 (PMC7725798; doi:10.3389/fbioe.2020.588925)
Supplement: Supplementary file 1 [file Data_Sheet_1.PDF]

Supplementary Material

1 Supplementary Tables

Table S1. Calibrated and generic muscle musculoskeletal parameters for the high functioning subject. The generic model used the same musculoskeletal parameters for both legs.

	High Functioning Subject								
	Calibrated						Generic		
	Paretic Leg			Non-paretic Leg			Non-Paretic/Paretic Leg		
**Muscle**	**Lmo**	**Lts**	**Fmax**	**Lmo**	**Lts**	**Fmax**	**Lmo**	**Lts**	**Fmax**
Addbrev	0.11	0.04	671.58	0.10	0.03	800.85	0.11	0.04	625.82
Addlong	0.11	0.12	1051.62	0.11	0.12	1049.67	0.11	0.13	916.80
AddmagDist	0.18	0.09	570.09	0.18	0.09	568.23	0.17	0.08	597.30
AddmadIsch	0.17	0.23	618.88	0.17	0.22	606.53	0.15	0.21	597.30
AddmagMid	0.13	0.05	781.25	0.14	0.05	757.47	0.13	0.05	597.30
AddmagProx	0.10	0.04	998.66	0.08	0.04	1330.57	0.11	0.04	597.30
Bflh	0.13	0.33	1179.11	0.14	0.30	1094.37	0.10	0.32	1313.18
Bsfh	0.11	0.11	656.39	0.12	0.12	583.59	0.11	0.10	557.11
Edl	0.08	0.41	998.26	0.07	0.41	1065.00	0.07	0.39	603.50
Fdl	0.05	0.42	473.51	0.05	0.40	495.49	0.05	0.40	423.18
Gaslat	0.07	0.41	1666.21	0.06	0.45	1779.68	0.06	0.40	1575.06
Gasmed	0.05	0.45	3557.91	0.06	0.43	3162.63	0.05	0.42	3115.51
Glmax1	0.16	0.06	1157.24	0.19	0.05	981.63	0.15	0.05	983.78
Glmax2	0.21	0.09	1256.13	0.20	0.08	1297.65	0.16	0.07	1406.05
Glmax3	0.23	0.09	770.68	0.23	0.08	752.08	0.17	0.07	947.75
Glmed1	0.08	0.06	1256.73	0.09	0.07	1060.98	0.07	0.06	1093.47
Glmed2	0.08	0.07	799.85	0.08	0.06	807.94	0.07	0.07	765.09
Glmed3	0.08	0.05	902.93	0.09	0.05	853.50	0.08	0.05	871.20
Glmin1	0.08	0.02	310.21	0.06	0.02	383.68	0.07	0.02	374.05
Glmin2	0.06	0.03	399.22	0.07	0.03	383.98	0.05	0.03	394.82
Glmin3	0.04	0.05	636.46	0.04	0.05	652.36	0.04	0.05	446.77
Iliacus	0.10	0.11	1355.70	0.10	0.10	1258.78	0.11	0.10	1021.14
Perbrev	0.05	0.16	572.09	0.05	0.16	598.42	0.05	0.16	521.20
Perlong	0.05	0.35	1173.99	0.05	0.35	1176.27	0.05	0.35	1115.37

Psoas	0.06	0.17	3321.26	0.09	0.13	2198.54	0.12	0.10	1426.79
Recfem	0.10	0.44	1960.12	0.09	0.47	2233.32	0.07	0.44	2191.74
Semimem	0.12	0.32	1449.04	0.10	0.32	1742.73	0.07	0.34	2200.99
Semiten	0.23	0.27	586.55	0.23	0.29	583.87	0.19	0.24	591.30
Soleus	0.06	0.31	5021.90	0.05	0.32	6396.59	0.05	0.30	6194.84
Tibant	0.07	0.27	1432.49	0.07	0.28	1460.27	0.07	0.26	1227.45
Tibpost	0.04	0.30	2099.04	0.04	0.32	1953.01	0.04	0.30	1730.15
Vasint	0.17	0.19	1172.28	0.11	0.23	1829.14	0.10	0.20	1697.36
Vaslat	0.14	0.22	4271.12	0.09	0.25	6644.75	0.10	0.22	5148.77
Vasmed	0.15	0.20	2099.76	0.10	0.23	3237.57	0.10	0.20	2747.82

Table S2. Calibrated and generic muscle musculoskeletal parameters for the low functioning subject. The generic model used the same musculoskeletal parameters for both legs.

	Low Functioning Subject								
	Calibrated						Generic		
	Paretic Leg			Non-paretic Leg			Non-Paretic/Paretic Leg		
**Muscle**	**Lmo**	**Lts**	**Fmax**	**Lmo**	**Lts**	**Fmax**	**Lmo**	**Lts**	**Fmax**
Addbrev	0.11	0.04	763.60	0.09	0.03	909.59	0.10	0.03	625.82
Addlong	0.08	0.15	1682.29	0.10	0.12	1328.75	0.10	0.13	916.80
AddmagDist	0.19	0.09	605.18	0.19	0.09	594.93	0.17	0.08	597.30
AddmadIsch	0.16	0.23	688.89	0.16	0.23	686.06	0.15	0.21	597.30
AddmagMid	0.14	0.05	806.24	0.14	0.05	789.81	0.13	0.04	597.30
AddmagProx	0.10	0.04	1120.89	0.11	0.04	1049.63	0.10	0.04	597.30
Bflh	0.10	0.36	1678.11	0.10	0.35	1722.22	0.10	0.32	1313.18
Bsfh	0.11	0.10	711.98	0.11	0.11	697.80	0.11	0.10	557.11
Tfl	0.09	0.45	548.75	0.07	0.47	684.60	0.09	0.44	411.21
Gaslat	0.06	0.44	2068.08	0.05	0.45	2604.91	0.07	0.42	1575.06
Gasmed	0.05	0.47	3783.39	0.06	0.47	3581.18	0.06	0.45	3115.51
Glmax1	0.18	0.05	1102.61	0.18	0.05	1145.33	0.14	0.05	983.78
Glmax2	0.20	0.08	1442.16	0.19	0.08	1515.78	0.15	0.06	1406.05
Glmax3	0.22	0.08	880.45	0.21	0.08	903.29	0.16	0.07	947.75
Glmed1	0.08	0.07	1370.88	0.07	0.06	1451.70	0.07	0.05	1093.47
Glmed2	0.08	0.07	944.37	0.07	0.07	981.86	0.07	0.06	765.09
Glmed3	0.08	0.04	1088.26	0.08	0.05	1052.60	0.07	0.04	871.20
Glmin1	0.07	0.02	371.14	0.07	0.02	364.95	0.06	0.02	374.05
Glmin2	0.06	0.03	493.51	0.06	0.03	460.47	0.05	0.02	394.82
Glmin3	0.04	0.05	844.87	0.04	0.05	757.83	0.04	0.05	446.77
Iliacus	0.09	0.09	1605.50	0.07	0.10	1971.38	0.10	0.09	1021.14
Perbrev	0.05	0.18	621.79	0.05	0.17	630.91	0.05	0.16	521.20
Perlong	0.05	0.36	1244.77	0.05	0.37	1241.69	0.06	0.36	1115.37
Psoas	0.10	0.10	2203.52	0.11	0.10	1925.07	0.11	0.09	1426.79
Recfem	0.09	0.46	2537.25	0.09	0.44	2532.54	0.07	0.44	2191.74
Semimem	0.10	0.33	1956.25	0.09	0.33	2231.42	0.07	0.34	2200.99
Semiten	0.21	0.25	704.83	0.19	0.29	784.52	0.19	0.25	591.30
Soleus	0.05	0.33	7168.23	0.05	0.32	7455.26	0.05	0.31	6194.84
Tibant	0.07	0.28	1453.58	0.08	0.29	1438.47	0.08	0.27	1227.45
Tibpost	0.03	0.33	2433.49	0.03	0.32	2618.80	0.04	0.31	1730.15

Vasint	0.15	0.15	1448.43	0.17	0.17	1305.77	0.10	0.20	1697.36
Vaslat	0.15	0.20	4364.71	0.14	0.21	4610.85	0.10	0.22	5148.77
Vasmed	0.15	0.20	2249.66	0.15	0.19	2373.90	0.10	0.20	2747.82

Table S3. Average RMS errors between muscle activations produced from static optimization and those obtained from the EMG-driven model.

	High Functioning Subject						Low Functioning Subject					
	Paretic Leg			Non-paretic Leg			Paretic Leg			Non-paretic Leg		
**Muscle**	**SOGen**	**SOCal**	**EMGCal**	**SOGen**	**SOCal**	**EMGCal**	**SOGen**	**SOCal**	**EMGCal**	**SOGen**	**SOCal**	**EMGCal**
Addbrev	0.02	0.02	0	0.02	0.02	0	0.03	0.01	0	0.02	0.02	0
Addlong	0.03	0.02	0	0.06	0.02	0	0.05	0.02	0	0.05	0.02	0
AddmagDist	0.02	0.02	0	0.02	0.01	0	0.02	0.01	0	0.02	0.02	0
AddmadIsch	0.02	0.02	0	0.01	0.04	0	0.02	0.01	0	0.02	0.02	0
AddmagMid	0.02	0.02	0	0.02	0.02	0	0.02	0.01	0	0.02	0.02	0
AddmagProx	0.16	0.16	0	0.02	0.02	0	0.14	0.15	0	0.06	0.04	0
Bflh	0.23	0.21	0	0.18	0.11	0	0.02	0.02	0	0.25	0.23	0
Bsfh	0.33	0.24	0	0.26	0.07	0	0.29	0.07	0	0.41	0.30	0
Tfl	-	-	-	-	-	-	0.45	0.38	0	0.35	0.46	0
Edl	0.24	0.03	0	0.19	0.07	0	-	-	-	-	-	-
Fdl	0.01	0.02	0	0.42	0.39	0	-	-	-	-	-	-
Gaslat	0.09	0.03	0	0.43	0.31	0	0.04	0.03	0	0.09	0.08	0
Gasmed	0.17	0.09	0	0.11	0.04	0	0.21	0.08	0	0.20	0.13	0
Glmax1	0.04	0.14	0	0.05	0.08	0	0.04	0.05	0	0.07	0.12	0
Glmax2	0.08	0.13	0	0.09	0.15	0	0.06	0.05	0	0.07	0.15	0
Glmax3	0.02	0.04	0	0.02	0.02	0	0.02	0.02	0	0.03	0.04	0
Glmed1	0.23	0.28	0	0.32	0.11	0	0.13	0.12	0	0.14	0.09	0
Glmed2	0.14	0.21	0	0.07	0.06	0	0.13	0.11	0	0.13	0.24	0
Glmed3	0.24	0.27	0	0.15	0.14	0	0.25	0.11	0	0.24	0.21	0
Glmin1	0.10	0.04	0	0.17	0.09	0	0.02	0.03	0	0.08	0.14	0
Glmin2	0.11	0.04	0	0.09	0.12	0	0.02	0.06	0	0.08	0.12	0
Glmin3	0.08	0.06	0	0.07	0.04	0	0.03	0.11	0	0.15	0.16	0
Iliacus	0.18	0.05	0	0.28	0.07	0	0.16	0.04	0	0.22	0.19	0
Perbrev	0.08	0.02	0	0.08	0.04	0	0.07	0.04	0	0.14	0.11	0
Perlong	0.13	0.04	0	0.15	0.08	0	0.13	0.02	0	0.17	0.09	0
Psoas	0.17	0.03	0	0.20	0.03	0	0.39	0.10	0	0.40	0.42	0
Recfem	0.16	0.12	0	0.26	0.20	0	0.17	0.09	0	0.20	0.25	0
Semimem	0.22	0.22	0	0.21	0.14	0	0.10	0.09	0	0.05	0.06	0
Semiten	0.22	0.15	0	0.18	0.09	0	0.10	0.09	0	0.23	0.19	0
Soleus	0.38	0.25	0	0.08	0.07	0	0.02	0.06	0	0.49	0.30	0
Tibant	0.23	0.32	0	0.26	0.12	0	0.45	0.06	0	0.32	0.20	0

Tibpost	0.03	0.04	0	0.09	0.16	0	0.32	0.34	0	0.26	0.16	0
Vasint	0.13	0.11	0	0.07	0.05	0	0.02	0.05	0	0.12	0.06	0
Vaslat	0.12	0.05	0	0.14	0.08	0	0.13	0.11	0	0.11	0.05	0
Vasmed	0.15	0.09	0	0.04	0.03	0	0.02	0.02	0	0.10	0.04	0
**Average**	0.13	0.10	0	0.14	0.09	0	0.12	0.077	0	0.16	0.14	0

Table S4. Average RMS errors between muscle produced joint moments from SOGen and EMGCal relative to SOCal across all trials

Joint Moments	High Functioning Subject						Low Functioning Subject					
	Paretic Leg			Non-paretic Leg			Paretic Leg			Non-paretic Leg		
	**SOGen**	**SOCal**	**EMGCal**	**SOGen**	**SOCal**	**EMGCal**	**SOGen**	**SOCal**	**EMGCal**	**SOGen**	**SOCal**	**EMGCal**
Hip flexion	2.29	0	5.12	3.60	0	6.65	1.00	0	7.03	0.71	0	5.46
Hip adduction	2.85	0	5.53	2.04	0	7.31	0.88	0	4.83	1.03	0	5.97
Hip rotation	0.42	0	2.00	0.49	0	2.60	0.78	0	1.74	0.43	0	2.17
Knee flexion	1.57	0	5.15	2.20	0	5.97	9.49	0	7.68	1.75	0	4.75
Ankle rotation	0.36	0	6.47	0.70	0	5.73	2.60	0	4.33	0.84	0	6.18
Ankle eversion	1.18	0	1.86	0.49	0	2.91	2.17	0	1.15	1.02	0	3.31

2 Overview of EMG-driven Modeling Methods

Nomenclature:

f_l	Active force-length function
$b_1, b_2 \dots, b_9$	Coefficients
c_1, c_2, c_3	Constants
$g_1, g_2 \dots, g_5$	Constants
d	Electromechanical time delay
f_v	Force-velocity function
θ	Joint angle
$\dot{\theta}$	Joint angular velocity
r	Moment arm
a	Muscle activation
τ_{act}	Muscle activation time constant
τ_{deact}	Muscle deactivation time constant
e	Muscle excitation
α	Muscle pennation angle
M	Muscle produced moment about a given joint
l^{MT}	Muscle-tendon length
v^{MT}	Muscle-tendon velocity
u	Neural activation
$\tilde{l}^M$	Normalized muscle fiber length
$\tilde{v}^M$	Normalized muscle fiber velocity
l_o^M	Optimal fiber length
f_p	Passive force-length function
F_o^M	Peak isometric force
l_s^T	Tendon slack length
t	Time
i	Time frame
Δt	Time interval

To develop our EMG-driven models, we used a Hill-type muscle model with a rigid tendon (Delp et al., 1990) where the muscle-tendon lengths, velocities, and moment arms were approximated by polynomial functions of model generalized coordinates and their first derivatives (Menegaldo et al., 2004; Sartori et al., 2012).

The moments produced by each muscle can be calculated by the following equation:

$$M = r \cdot F_o^M \cdot [a \cdot f_l\left(\tilde{l}^M(t)\right) \cdot f_v\left(\tilde{v}^M(t)\right) + f_p\left(\tilde{l}^M(t)\right)]cos\alpha$$
$$0 \le a(t) \le 1 \qquad (1)$$

$$0.3 < \tilde{l}^M(t) < 1.3$$

$$-1 < \tilde{v}^M(t) < 1$$

$\tilde{l}^M$ and $\tilde{v}^M$ are calculated neglecting tendon compliance using the following equations:

$$\tilde{l}^M = \frac{l^{MT} - l_s^T}{l_o^M \cdot cos\alpha} \tag{2}$$

$$\tilde{v}^M = \frac{v^{MT}}{10 \cdot l_o^M} \tag{3}$$

Our EMG-driven model requires specification of five parameter values d, l_o^M, l_s^T, F_o^M, and α and four variables a, l^{MT}, v^{MT}, and r. We describe how to calculate a, l^{MT}, v^{MT}, and r below.

To calculate muscle activations, a first order differential equation (He et al., 1991) is discretized to find neural activations from muscle excitations as follows:

$$u_i = \frac{2\Delta t(c_1 e(t_i - d) + c_2)e(t_i - d) + 4u_{i-1} - u_{i-2}}{2\Delta t(c_1 e(t_i - d) + c_2) + 3} \tag{4}$$

The constants c_1 and c_2 are defined as:

$$c_1 = \frac{1}{\tau_{act}} - \frac{1}{\tau_{deact}} \tag{5}$$

$$c_2 = \frac{1}{\tau_{deact}} \tag{6}$$

where $\tau_{deact} = 4\tau_{act}$ based on the ratio reported in literature (Zajac, 1989; Thelen, 2003; De Groote et al., 2012; Millard et al., 2013). A nonlinear function is then used to find muscle activations from neural activations as follows (Manal and Buchanan, 2003a).

$$a_i = (1 - c_3)u_1 + c_3 \left[\frac{g_1}{g_2(u_1 + g_3)^{g_4} + g_5} + 1 \right] \quad (7)$$

where c_3 can vary from forming a linear (0) to a highly nonlinear (0.35) relationship. Constants g_1 to g_5 have values of -7.623, 29.280, 0.884, 17.227, and 4.108 (Manal and Buchanan, 2003b).

The variables l^{MT}, v^{MT}, and r are calculated using polynomial functions of the joint angles and velocities (Menegaldo et al., 2004; Sartori et al., 2012). For muscles that span one degree of freedom (DOF), the l^{MT} can be approximated using the following cubic polynomial equations (An et al., 1984):

$$l^{MT} = b_0 + b_1\theta + b_2\theta^2 + b_3\theta^3 \quad (8)$$

v^{MT} and r can be computed accordingly as follows:

$$v^{MT} = \frac{dl^{MT}}{dt} = b_1\dot{\theta} + 2b_2\theta\dot{\theta} - 3b_3\theta^2\dot{\theta} \quad (9)$$

$$r = -\frac{\partial l^{MT}}{\partial \theta} = -b_1 - 2b_2\theta - 3b_3\theta^2 \quad (10)$$

For muscles that span two DOFs, these equations are extended as follows:

$$l^{MT} = b_0 + b_1\theta_1 + b_2\theta_2 + b_3\theta_1\theta_2 + b_4\theta_1^2 + b_5\theta_2^2 + b_6\theta_1^2\theta_2 + b_7\theta_1\theta_2^2 + b_8\theta_1^3 + b_9\theta_2^3 \quad (11)$$

$$\begin{aligned} v^{MT} = {} & b_1\dot{\theta}_1 + b_2\dot{\theta}_2 + b_3(\dot{\theta}_1\theta_2 + \theta_1\dot{\theta}_2) + 2b_4\theta_1\dot{\theta}_1 + 2b_5\theta_2\dot{\theta}_2 + b_6(2\theta_1\dot{\theta}_1\theta_2 + \theta_1^2\dot{\theta}_2) \\ & + b_7(\dot{\theta}_1\theta_2^2 + 2\theta_1\theta_2\dot{\theta}_2) + 3b_8\theta_1^2\dot{\theta}_1 + 3b_9\theta_2^2\dot{\theta}_2 \end{aligned} \quad (12)$$

$$r_1 = -\frac{\partial l^{MT}}{\partial \theta_1} = -b_1 - b_3\theta_2 - 2b_4\theta_1 - 2b_6\theta_1\theta_2 - b_7\theta_2^2 - 3b_8\theta_1^2 \quad (13)$$

$$r_2 = -\frac{\partial l^{MT}}{\partial \theta_2} = -b_2 - b_3\theta_1 - 2b_5\theta_2 - b_6\theta_1^2 - 2b_7\theta_1\theta_2 - 3b_9\theta_2^2 \tag{14}$$

Similarly, for muscles that span three or four DOFs, Equations (8), (9), and (10) can be extended by adding additional terms for joint angles and velocities.

References

An, K. N., Takahashi, K., Harrigan, T. P., and Chao, E. Y. (1984). Determination of Muscle Orientations and Moment Arms. *J. Biomech. Eng.* 106, 280–282. doi:10.1115/1.3138494.

De Groote, F., Demeulenaere, B., Swevers, J., De Schutter, J., and Jonkers, I. (2012). A physiology-based inverse dynamic analysis of human gait using sequential convex programming: a comparative study. *Comput. Methods Biomech. Biomed. Engin.* 15, 1093–1102. doi:10.1080/10255842.2011.571679.

Delp SL, Loan JP, Hoy MG, Zajac FE, Topp EL, Rosen JM. An interactive graphics-based model of the lower extremity to study orthopaedic surgical procedures. IEEE Trans Biomed Eng. 1990; 37: 757–67. https://doi.org/10.1109/10.102791

He J, Levine WS, Loeb GE. Feedback gains for correcting small perturbations to standing posture. IEEE Trans Automat Contr. 1991; 36: 322–332. https://doi.org/10.1109/9.73565

Manal, K., and Buchanan, T. S. (2003a). A one-parameter neural activation to muscle activation model: estimating isometric joint moments from electromyograms. *J. Biomech.* 36, 1197–1202.

Manal, K., and Buchanan, T. S. (2003b). A one-parameter neural activation to muscle activation model: Estimating isometric joint moments from electromyograms. *J. Biomech.* 36, 1197–1202. doi:10.1016/S0021-9290(03)00152-0.

Menegaldo, L. L., De Toledo Fleury, A., and Weber, H. I. (2004). Moment arms and musculotendon lengths estimation for a three-dimensional lower-limb model. *J. Biomech.* 37, 1447–1453. doi:10.1016/j.jbiomech.2003.12.017.

Millard, M., Uchida, T., Seth, A., and Delp, S. L. (2013). Flexing computational muscle: Modeling and simulation of musculotendon dynamics. *J. Biomech. Eng.* 135. doi:10.1115/1.4023390.

Sartori, M., Reggiani, M., van den Bogert, A. J., and Lloyd, D. G. (2012). Estimation of musculotendon kinematics in large musculoskeletal models using multidimensional B-splines. *J. Biomech.* 45, 595–601. doi:10.1016/j.jbiomech.2011.10.040.

Thelen, D. G. (2003). Adjustment of muscle mechanics model parameters to simulate dynamic contractions in older adults. *J. Biomech. Eng.* 125, 70–77. doi:10.1115/1.1531112.

Zajac, F. E. (1989). Muscle and tendon: properties, models, scaling, and application to biomechanics and motor control. *Crit. Rev. Biomed. Eng.* 17, 359–411.

3 Detailed Flowcharts

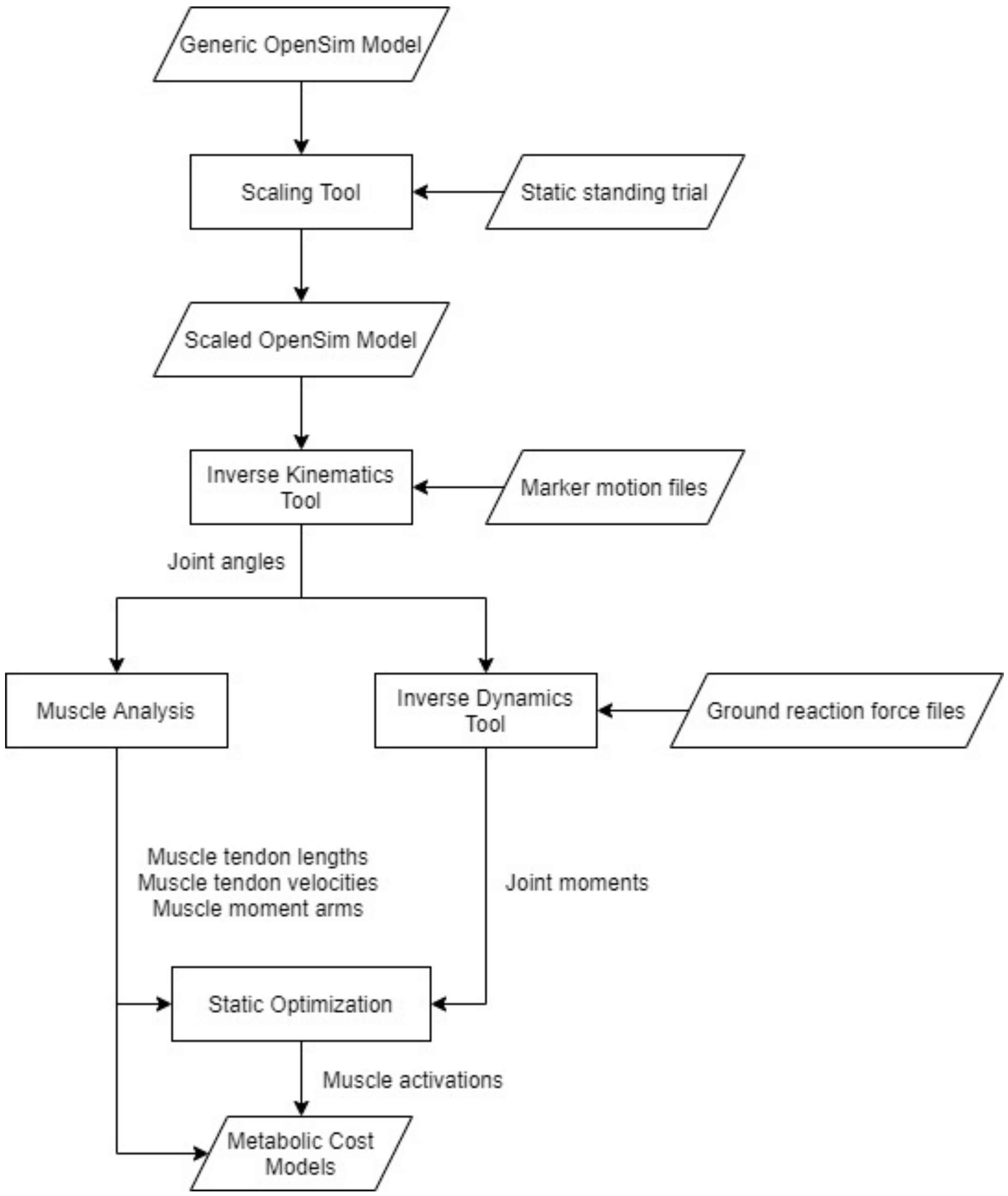

Figure S1. Flowchart for the SOGen method.

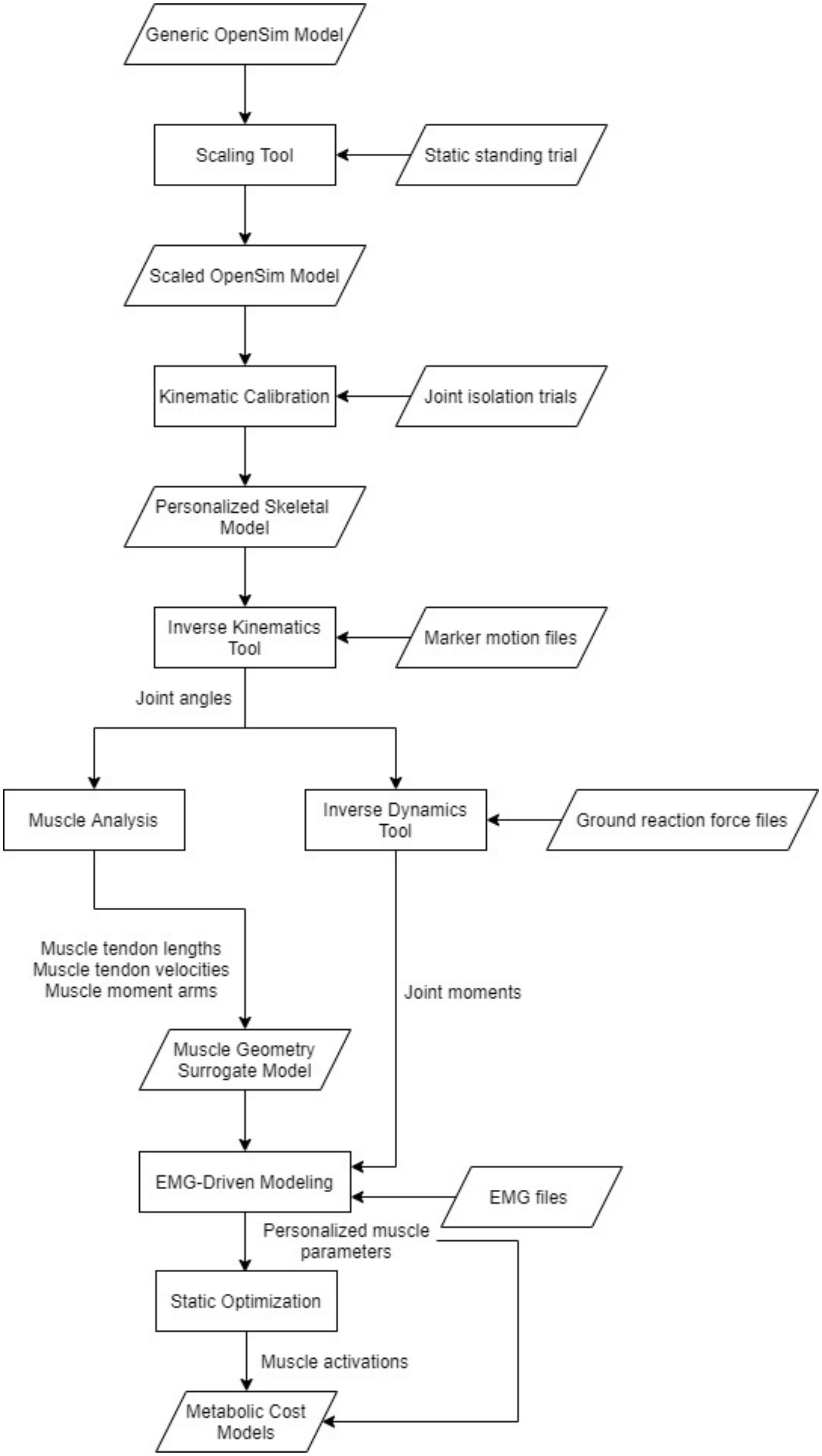

Figure S2. Flowchart for the SOCal method.

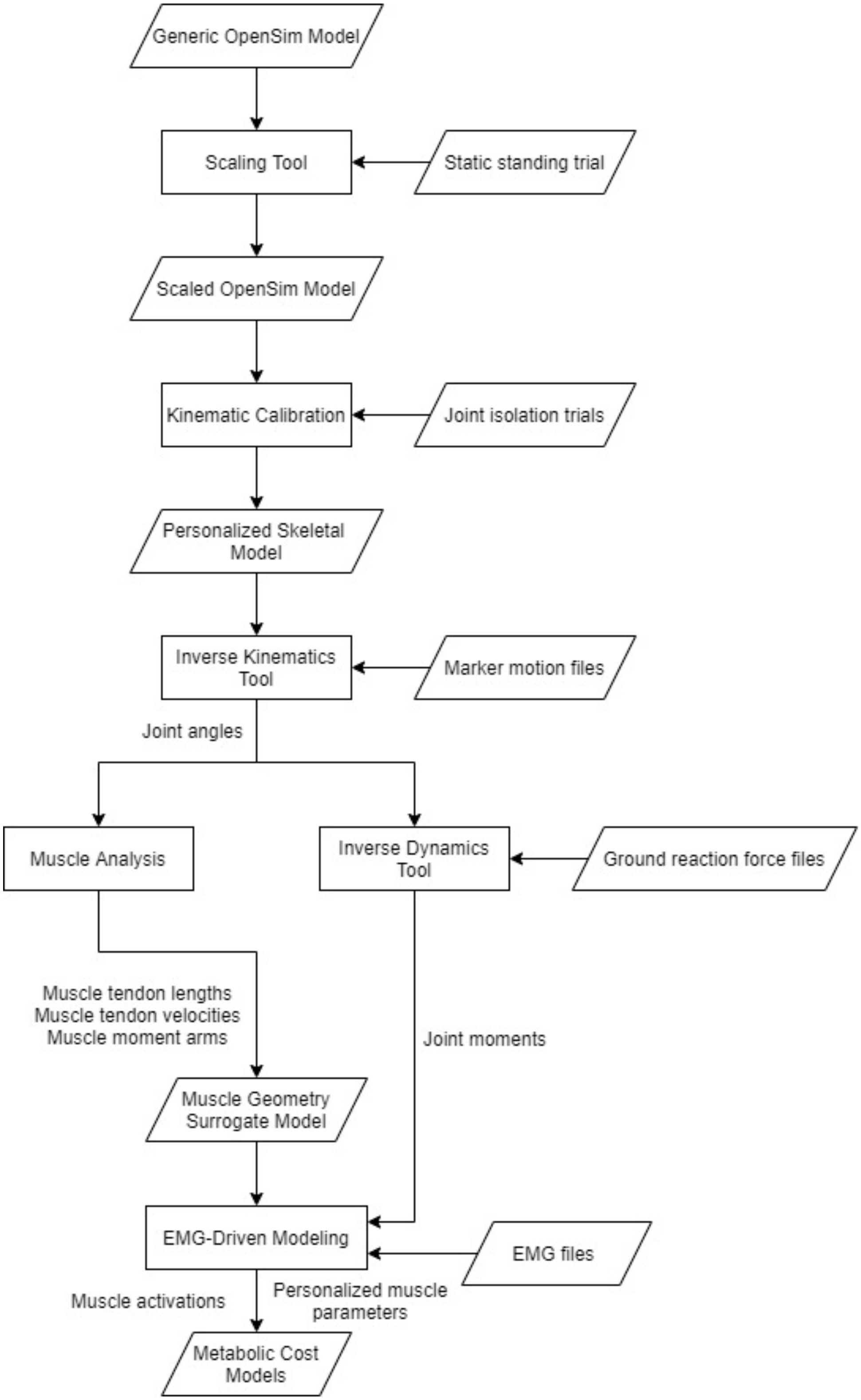

Figure S3. Flowchart for the EMGCal method.
